# Large-scale conversational data auditability via cryptographic commitments and hash-chained audit-ledger verification

**DOI:** 10.3389/frai.2026.1929127

**Published:** 2026-10-01

**Authors:** Iván Ortiz-Gárces, Pablo Palacios, Washington Loza-Herrera, William Villegas-Ch

**Affiliations:** 1 Escuela de Ingeniería en Ciberseguridad, Facultad de Ingeniería y Ciencias Aplicadas, Universidad de Las Américas, Quito, Ecuador; 2 Escuela de Informática y Telecomunicaciones, Universidad Diego Portales, Santiago, Chile; 3 Research Group in Software Production Models (GrIMPSoft), Department of Computer Science, Universidad de las Fuerzas Armadas ESPE, Sangolquí, Ecuador; 4 Department of Computer Science, Universidad Rey Juan Carlos, Madrid, Spain

**Keywords:** blockchain, cryptographic commitments, LLM auditability, Merkle verification, privacy-preserving traceability

## Abstract

The auditability of large language model-based systems requires verifiable evidence without exposing the sensitive content of interactions. This article proposes a hash-chained audit-ledger framework to transform public LLM conversations into canonical off-chain units, generate cryptographic commitments using SHA-256 and HMAC-SHA-256, build a lightweight ledger with Merkle roots, and verify integrity against induced tampering. The study integrates three heterogeneous sources, LMSYS-Chat-1M, WildChat, and Chatbot Arena, and produces 3,971,887 auditable units. In the full-corpus HMAC-SHA-256 experiment, the 11,899.55 MiB normalized repository was represented by 1,473.44 MiB of hash-only audit records, 2.03 MiB of ledger block headers, and 380.52 MiB for the operational ledger plus membership structures required for record-level verification. Five induced tampering categories, Merkle and decision-hash integrity, hash-chain linkage integrity, block ordering integrity, membership completeness, and dataset-membership consistency, were detected without false acceptance; a separate clean-ledger baseline validated the untampered ledger with zero error signals. Load experiments showed near-linear growth of mean total pipeline time with respect to the number of audit records. Operational ledger-stage throughput increased across the intermediate workloads but declined at the largest evaluated load, while full record-set verification throughput remained comparatively stable up to 250,000 interactions before decreasing in the 500,000-interaction scenario. This contribution demonstrates that the traceability of decisions generated by LLM can be formulated as a measurable, private, and cryptographically verifiable experimental chain. The framework provides a documented numerical-audit basis for independent consistency assessment, regulatory compliance, and forensic analysis when ledger states are evaluated against preserved artifacts, trusted checkpoints, or externally anchored commitments.

## 1 Introduction

Large language model (LLM) systems are involved in tasks where simply explaining a decision is no longer enough: it is also necessary to know how it was produced, under what interaction, and with what possibility of independent verification. This requirement arises when AI governance attempts to translate principles of reliability into evaluable mechanisms, yet a gap remains among regulation, technical auditing, and computable evidence. Laux et al. (2024) warns that normative trust should not be confused with risk acceptability; Gorwa and Veale (2024) show that model intermediaries introduce new liability problems; and Baldwin (2025) demonstrates that bias audits in LLMs require reproducible procedures. In parallel, Du et al. (2024) reinforce the importance of auditing the integrity of outsourced data, which is critical when LLM interactions are stored outside traditional institutional infrastructures.

Privacy-aware traceability offers a promising, albeit incomplete, path. Abid et al. (2025) shows that interorganizational processes can be traced without exposing full identities; Zhou (2025) proposes an auditable blockchain architecture for sensitive operations; and Sutradhar et al. (2024) combines blockchain, secure transmission, and data protection in medical contexts. These works confirm that separating sensitive data from verifiable evidence is feasible, but they do not address the case of conversations with LLM, where the auditable object is not predefined. Unlike a transaction, a conversation can contain turns, multiple responses, comparisons, variable lengths, and heterogeneous metadata. Therefore, the challenge is not recording events on a chain but rather constructing an auditable canonical representation first.

This work addresses this gap. The central premise is that the auditability of decisions generated by LLM requires a hybrid architecture: conversational data remains off-chain, while the chain maintains verifiable cryptographic commitments. Blockchain is not used as a content repository or as an independent proof of original model ownership, but rather as a layer for post-commitment integrity, provenance traceability, and tamper-evident validation relative to preserved ledger checkpoints. The fundamental question is whether public and heterogeneous conversations can be transformed into auditable and comparable units, whether these units can be protected using SHA-256 or HMAC-SHA-256 without losing verifiability, and whether the ledger maintains sufficient efficiency to operate at scale.

To address this problem, an experimental framework was designed that integrates canonical preparation, audit trail generation, ledger construction, cryptographic verification, tamper simulation, privacy assessment, and scalability testing. The methodological contribution lies in shifting auditing from full-text to a hash-only canonical ledger that preserves traceability without publishing prompts or complete responses. This allows evaluation of alteration detection, storage reduction, HMAC cost, and behavior under load and block-size variations.

The results anticipate the relevance of this approach. From LMSYS-Chat-1M, WildChat, and Chatbot Arena, 3,971,887 auditable units were generated, demonstrating that public conversational sources can become an extensive off-chain database for post-verification. In the full-corpus HMAC-SHA-256 experiment, the 11,899.55 MiB normalized repository was transformed into 1,473.44 MiB of hash-only audit records. The corresponding lightweight ledger required 2.03 MiB for block headers alone and 380.52 MiB when the membership mapping needed for record-level verification was included. These findings suggest that the auditability of LLMs can be formulated as a measurable experimental problem, in which privacy, integrity, and scalability are evaluated within a single chain of evidence.

The work is organized as follows. The Materials and Methods section formalizes the audit records, blocks, Merkle roots, SHA-256/HMAC-SHA-256 commits, and experimental metrics. The Results section presents canonical preparation, storage reduction, cryptographic validation, tamper detection, operational performance, and scalability. The Discussion section interprets the framework's scope as a contribution to independent audits, and the Conclusions section emphasizes regulatory compliance and verifiable traceability of generative decisions.

## 2 Literature review

Recent literature on AI auditing has advanced rapidly. Still, a central tension remains: most proposals define what should be audited, while few show how to produce verifiable, reproducible, and privacy-preserving evidence at the interaction scale. Mökander et al. (2024) offers a useful perspective by distinguishing among governance, model, and application audits; however, their contribution is primarily conceptual and does not address the granular traceability of LLM-generated conversations. Along similar lines, Laine et al. (2024), Schiff et al. (2024), and Díaz-Rodríguez et al. (2023) show that ethical auditing and trustworthy AI require operational evidence, not just normative principles. The problem is that this evidence is often framed as an institutional requirement, without a technical layer capable of transforming usage events into cryptographically verifiable, comparable, and auditable objects.

Work specifically focused on LLMs is beginning to close this gap, albeit only partially. Sgantzos et al. (2023) applies the logic of triple-entry accounting to LLM auditing, providing a solid insight into immutable records and shared responsibility. Li et al. (2026) advances toward a hash-chain architecture for auditable LLMs, bringing the debate closer to concrete cryptographic commitments. However, these approaches tend to focus on the audit framework or the sequence of commitments, rather than demonstrating whether heterogeneous public conversations can be normalized into a sufficiently comprehensive off-chain basis to support subsequent verification. Sachan and Liu (2024) also demonstrate the value of blockchain for auditing decisions supported by explainable and generative AI. Still, its legal domain reinforces a common limitation: traceability is discussed in relation to relatively structured decisions, not massive, variable, and multi-turn conversational interactions.

The blockchain literature offers more mature mechanisms for integrity, immutability, and reduced data exposure, but these do not always align with the specific problem faced by LLMs. Davies (2024) shows that Merklized transactions can improve scalability and privacy, supporting an architecture in which the ledger preserves commitments rather than sensitive content. The design also relates to the broader family of append-only transparency systems. Certificate Transparency uses Merkle-tree logs to make certificate issuance publicly auditable (Laurie et al., 2013); Trillian generalizes verifiable logs and maps for arbitrary transparency applications (Trillian Project, 2026); and OpenTimestamps anchors document-existence proofs through hash commitments that can later be independently verified (OpenTimestamps, 2026). These systems clarify the distinction between a hash chain that is internally consistent and an externally anchored or publicly auditable log. The present work adopts the former as a lightweight experimental audit ledger and treats external anchoring as an operational deployment control when stronger adversarial assumptions are required. Jia et al. (2024), Ma and Zhang (2024), Chen et al. (2024), Hu et al. (2024), and Wang et al. (2026) delve into privacy, regulatory compliance, access control, ZK-Rollups, interoperability, and cross-chain auditing. Taken together, these studies demonstrate that blockchain can operate as a verification and traceability layer without publishing complete data. However, their focus is typically on financial transactions, healthcare, identity, or organizational data; therefore, they do not directly address the challenge of converting conversational LLM content into canonical, auditable units with hashes, post-commitment provenance evidence, and verifiable performance metrics.

Approaches to private traceability in applied domains reinforce this interpretation. Li et al. (2024) proposes privacy-preserving traceability for supply chains, while Ullah et al. (2024) applies blockchain to access auditing in healthcare. Both are relevant because they separate verifiable records from sensitive content, but their auditable units originate from events that are relatively well defined by the system. In contrast, decisions generated by LLMs require a more complex preliminary stage: identifying, normalizing, and committing interactions that can vary in length, structure, participants, models, and levels of granularity. This difference is substantial because auditability depends not only on the ledger but also on the quality of the canonical object to which it is anchored.

The gap lies not in the absence of blockchain, hashes, or auditing principles, but in the experimental integration between the three levels. Normative literature demands accountability, blockchain literature offers integrity mechanisms, and emerging literature on LLM systems proposes specific audits. Still, few contributions build the complete bridge: canonical off-chain preparation, SHA-256 or HMAC-SHA-256 commits, ledger construction, verification, tamper detection, storage reduction, and scalability. Therefore, an experimental framework focused on public LLM conversations offers novelty if it demonstrates that traceability can be maintained without exposing prompts or complete responses, and if its metrics allow for evaluating not only whether the system works, but also its cost, how much it reduces storage requirements, and how verifiable it remains in the face of tampering.

## 3 Materials and methods

The methodology was designed to transform public conversations generated by LLMs into a standardized, auditable representation that enables lightweight hash-chained ledger verification and traceability. The methodological framework integrates data preparation, auditable record generation, ledger construction, integrity validation, and experimental evaluation into a unified processing flow.

Figure 1 shows the general architecture of the proposed audit framework. The process begins with the acquisition of public conversational datasets that contain prompt-response interactions and comparative records across models. These sources are then transformed into a common canonical representation that provides a homogeneous structure for subsequent audit stages. The normalized conversational units are then converted into auditable, privacy-aware authenticated commitment records that serve as input to the lightweight hash-chained audit-ledger construction process.

**Figure 1:**
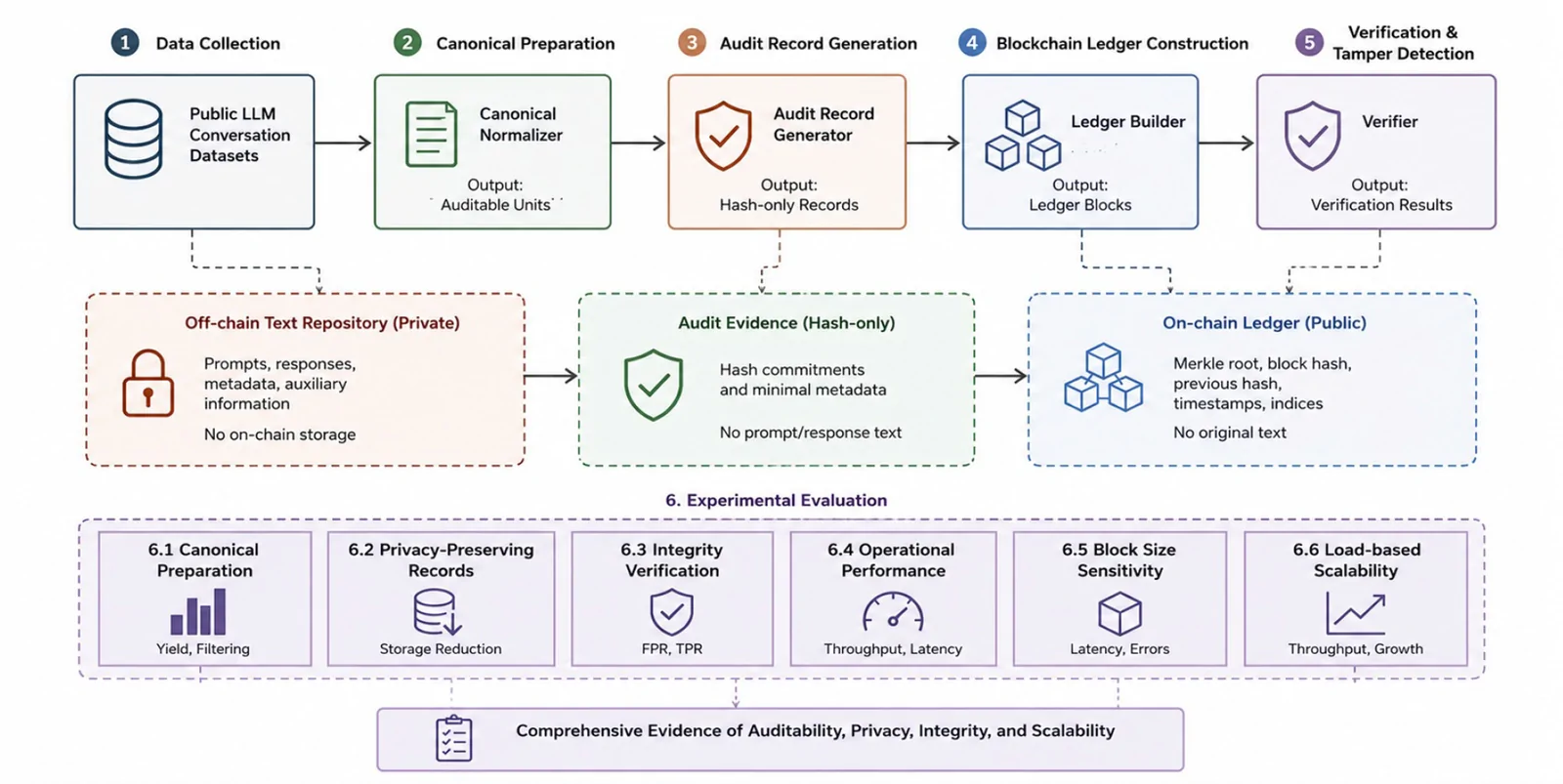
Overall architecture of the privacy-preserving hash-chained audit-ledger framework.

Once the ledger is built, cryptographic verification procedures are applied to assess the preservation of record integrity and the system's ability to detect unauthorized modifications relative to preserved or externally anchored reference states. Finally, the framework undergoes a series of controlled experiments to analyze the efficiency of the canonical transformation process, the generation of privacy-aware auditable records, integrity validation, operational performance, block-size sensitivity, and scalability behavior under different load levels.

The complete methodological flow can be represented by the transformation sequence shown in Equation 1:


$$\begin{array}{l} D\to A\to R\to B\to V \end{array}$$ (1)

where *D* represents the original conversational datasets, *A* the normalized auditable units, *R* the privacy-aware auditable records, *B* the generated lightweight hash-chained audit ledger, and *V* the verification and evaluation procedures applied to the ledger.

### 3.1 Canonical preparation of public LLM conversations

The construction of verifiable cryptographic evidence requires a unique structural representation of each audited observation before any serialization, hashing, or cryptographic aggregation. However, the conversational sources considered in this study do not share a common observational unit. LMSYS-Chat-1M and WildChat represent interactions as prompt–response sequences distributed across conversations of variable length, whereas Chatbot Arena stores pairwise comparisons between responses generated by different models for the same prompt, together with human preference information. Consequently, operationally equivalent interactions may generate incompatible evidence if processed directly from their original representations. To preserve observational equivalence across heterogeneous conversational domains, all records were transformed into a common, auditable structure before cryptographic processing.

Let *D* = {*D*_1_, *D*_2_, *D*_3_} be the set of conversational sources considered during the study. Each source contains observations with different structures, levels of granularity, and metadata conventions. To ensure a consistent representation across datasets, all observations were transformed into a canonical auditable unit defined in Equation 2:


$$\begin{array}{l} a_{i}=\langle id_{i},d_{i},c_{i},t_{i},p_{i},r_{i},m_{i},\tau_{i},L_{i},M_{i}\rangle \end{array}$$ (2)

where *id*_*i*_ uniquely identifies the audited observation, *d*_*i*_ denotes the source dataset, *c*_*i*_ corresponds to the original conversation, *t*_*i*_ indicates the relative position of the interaction within the conversational sequence, *p*_*i*_ and *r*_*i*_ represent the prompt and response respectively, *m*_*i*_ identifies the associated language model, τ_*i*_ stores the available temporal information, *L*_*i*_ preserves the language label, and *M*_*i*_ groups the auxiliary metadata retained during the transformation process.

Records originating from Chatbot Arena require an extended representation because multiple responses may be associated with the same prompt. For these observations, the auditable unit was defined in Equation 3:


$$\begin{array}{l} a_{i}^{cmp}=\langle \begin{array}{c} id_{i},d_{i},c_{i},t_{i},p_{i},\\ r_{i}^{A},r_{i}^{B},\\ m_{i}^{A},m_{i}^{B},\\ w_{i},j_{i},\tau_{i},L_{i},M_{i} \end{array}\rangle \end{array}$$ (3)

where $r_{i}^{A}$ and $r_{i}^{B}$ represent the responses generated by the compared models, $m_{i}^{A}$ and $m_{i}^{B}$ identify those models, *w*_*i*_ indicates the winning response selected during evaluation, and *j*_*i*_ preserves the information associated with the comparison procedure. This formulation allows conversational interactions and comparative evaluations to be represented within the same auditing framework while preserving the operational characteristics of the original observation.

After defining the canonical representation, all observations underwent structural validation to guarantee deterministic reconstruction. Records containing empty prompts, missing responses, incomplete mandatory fields, unidentified comparison outcomes, or non-reconstructible structures were discarded. Subsequently, model identifiers, language labels, temporal attributes, and auxiliary metadata were normalized to a common representation to eliminate source-specific encoding differences. Finally, all attributes were reorganized according to a deterministic ordering scheme, ensuring that equivalent observations produce identical serialized representations regardless of their dataset of origin.

### 3.2 Privacy-preserving audit record generation

Canonical preparation provides a homogeneous representation of audited interactions but does not eliminate the dependency between verifiable evidence and conversational content. Prompts, responses, and descriptive metadata remain embedded within the auditable units, making direct incorporation into a blockchain infrastructure impractical from both storage and privacy perspectives. To address this limitation, audit-record generation was formulated as a problem of preserving verifiability without storing conversational content directly within the ledger. Under this formulation, interactions are represented through reproducible cryptographic commitments derived from a deterministic serialization process.

Let S(x) denote the serialized representation of an arbitrary element *x*. The basic cryptographic commitment used throughout the auditing process is defined in Equation 4:


$$\begin{array}{l} h_{SHA}\left(x\right)=\text{SHA-}256\left(S\left(x\right)\right) \end{array}$$ (4)

where the hash function operates on the serialized representation rather than the original content. This formulation guarantees that any modification introduced to the audited attributes produces a corresponding alteration in the resulting commitment.

To evaluate authenticated evidence-generation scenarios, an alternative commitment based on HMAC-SHA-256 was also incorporated, as defined in Equation 5:


$$\begin{array}{l} h_{HMAC}\left(x\right)=\text{HMAC-SHA-}256\left(K,S\left(x\right)\right) \end{array}$$ (5)

where *K* represents a secret key used during commitment generation. This formulation preserves integrity while restricting unauthorized recomputation of commitments.

In the HMAC-SHA-256 configuration, key custody is treated as part of the authorized audit boundary rather than as a public verification primitive. The data controller or audit operator generates and stores *K* in a controlled environment. Independent auditors may receive either a verification service, a scoped derived key, or regenerated commitments produced under an agreed audit protocol. This design restricts unauthorized recomputation of content-derived commitments while preserving structural verification through Merkle roots and block hashes. If *K* is rotated, new records are committed under a new key identifier while previously generated commitments remain verifiable under the historical key. If *K* is compromised, confidentiality against offline recomputation is weakened for records produced with that key. However, compromise of *K* does not by itself make an internally stored ledger immutable. If an adversary controls all stored ledger artifacts and no trusted checkpoint exists, the adversary could modify records, recompute the affected Merkle roots, and rebuild subsequent block hashes into a new internally consistent chain. Therefore, the security claim of the present framework is tamper evidence relative to preserved audit artifacts, a trusted prior checkpoint, or an external anchoring mechanism that stores selected Merkle roots or block hashes outside the adversary's control. Full institutional key management, including hardware-backed storage, revocation workflows, multi-party custody, and periodic external anchoring, is an operational deployment control layered above the experimental framework, not a property supplied by hashing alone.

Individual commitments allow verification of isolated interaction components but do not provide a single representation of the audited observation. To preserve the interaction as an operational unit, an aggregate commitment referred to as the decision hash was defined in Equation 6:


$$\begin{array}{l} h\left(dec_{i}\right)=H(h\left(p_{i}\right)∥h\left(r_{i}\right)∥h\left(M_{i}\right)∥h\left(\mu_{i}\right)∥d_{i}∥m_{i}∥\tau_{i}) \end{array}$$ (6)

where *h*(*p*_*i*_), *h*(*r*_*i*_), *h*(*M*_*i*_), and *h*(μ_*i*_) correspond to the commitments associated with the prompt, response, metadata, and moderation or operational validation state, respectively, while *d*_*i*_, *m*_*i*_, and τ_*i*_ preserve provenance, model identity, and temporal information. The resulting commitment serves as the primary cryptographic representation of the audited interaction. The generic operator *H*(·) denotes the active commitment mode used in the experiment: SHA-256 in the unauthenticated configuration and HMAC-SHA-256 when authenticated privacy-preserving commitments are enabled. Thus, the aggregate decision hash is recomputed under the same mode as the component commitments, ensuring reproducibility across both experimental configurations.

To support large-scale auditing, the generated commitments were reorganized into compact hash-only audit records containing only the information required for reconstruction and verification. For standard conversational interactions, each record is defined in Equation 7:


$$\begin{array}{l} \rho_{i}=\langle \begin{array}{c} rid_{i},s_{i},d_{i},c_{i},t_{i},m_{i},\tau_{i},\\ L_{i},h\left(p_{i}\right),h\left(r_{i}\right),h\left(M_{i}\right),h\left(\mu_{i}\right),h\left(dec_{i}\right),alg \end{array}\rangle \end{array}$$ (7)

Thus, standard interaction records contain five cryptographic commitments: prompt, response, metadata, moderation or operational state, and aggregate decision hash. The non-cryptographic attributes preserve operational traceability, while the remaining fields correspond exclusively to commitments derived from the original interaction.

For comparative observations originating from Chatbot Arena, the auditable record is extended as defined in Equation 8:


$$\begin{array}{l} \rho_{i}^{cmp}=\langle \begin{array}{c} rid_{i},s_{i},d_{i},c_{i},t_{i},\\ m_{i}^{A},m_{i}^{B},w_{i},j_{i},\tau_{i},L_{i},\\ h\left(p_{i}\right),h\left(r_{i}^{A}\right),h\left(r_{i}^{B}\right),\\ h\left(M_{i}\right),h\left(\mu_{i}\right),h\left(dec_{i}\right),alg \end{array}\rangle \end{array}$$ (8)

Comparative records therefore contain six cryptographic commitments because the two candidate responses are committed separately. Here, $m_{i}^{A}$ and $m_{i}^{B}$ identify the compared models, *w*_*i*_ preserves the evaluation outcome, and *j*_*i*_ retains the information associated with the comparison procedure.

### 3.3 Hash-chained audit-ledger construction and integrity verification

The hash-only representation preserves the individual integrity of each audited interaction and eliminates the need to store conversational content in the verifiable infrastructure. However, the existence of millions of independent cryptographic commitments does not provide mechanisms for verifying the structural consistency of the entire evidence set. Under these conditions, ledger construction was formulated as a verifiable cryptographic aggregation problem, aimed at simultaneously preserving individual and collective integrity and enabling detection of tampering through Merkle structures and a hash-chained audit ledger.

Let *R* = {ρ_1_, ρ_2_, …, ρ_*n*_} be the ordered set of auditable records generated in the previous stage. The records were sequentially partitioned into configurable blocks and encapsulated using the structure defined in Equation 9:


$$\begin{array}{l} B_{j}=\langle j,prev_{j},MR_{j},n_{j},first_{j},last_{j},ts_{j},BH_{j}\rangle \end{array}$$ (9)

where *j* identifies the block, *prev*_*j*_ represents the cryptographic commit of the preceding block, *MR*_*j*_ corresponds to the Merkle root associated with the records contained in the block, *n*_*j*_ indicates the total number of observations stored, *first*_*j*_ and *last*_*j*_ identify the endpoints of the sequence, *ts*_*j*_ stores the timestamp of the construction, and *BH*_*j*_ represents the cryptographic identifier of the entire block.

Record aggregation was performed using Merkle trees constructed from the decision commits of each block. This strategy allows large volumes of evidence to be condensed into a single verifiable cryptographic representation, such that any modification to an individual record automatically alters the corresponding Merkle root. Formally, the aggregated evidence associated with block *B*_*j*_ is defined in Equation 10:


$$\begin{array}{l} MR_{j}=MerkleRoot\left(L_{j}\right) \end{array}$$ (10)

where *L*_*j*_ represents the set of cryptographic leaves derived from the records contained in the block.

Preserving global integrity also requires that any local modification produces observable effects throughout the chain. To achieve this, each block explicitly incorporates the cryptographic evidence of its predecessor via a hash-chaining mechanism. The identifier associated with each block is calculated using Equation 11:


$$\begin{array}{l} BH_{j}=\text{SHA-256}(j∥prev_{j}∥MR_{j}\\ \;∥n_{j}∥first_{j}\\ \;∥last_{j}∥ts_{j}) \end{array}$$ (11)

where ∥ represents the deterministic concatenation of the elements that make up the block header. Because the calculation simultaneously incorporates the Merkle root and the identifier of the preceding block, any alteration introduced in an individual record is propagated to subsequent blocks, making the alteration detectable when the reconstructed chain is compared with a preserved copy, trusted checkpoint, or externally anchored digest.

In addition to the main hash-chained audit-ledger structure, an auxiliary mechanism called membership mapping was implemented. This mechanism is responsible for maintaining the correspondence between each auditable record, its associated Merkle leaf, the assigned block, and its position in the chain. This structure allows for the reconstruction of proofs of membership and the location of specific records without traversing the entire ledger sequentially.

Integrity validation was performed by independently reconstructing the stored cryptographic evidence. The procedure begins by regenerating the Merkle leaves and recalculating the roots for each block. Subsequently, the blocks' cryptographic identifiers are reconstructed, and the consistency of links between consecutive blocks is verified. Finally, the associations stored in the membership mapping enable verification of individual records' membership and validation of the correspondence between the audited evidence and the structure in which it was incorporated.

The combination of Merkle aggregation, hash chaining, and membership validation transforms massive sets of audited conversations into a verifiable infrastructure capable of detecting local alterations and structural manipulations using cryptographic evidence. This capability is conditional on comparison against a trusted state, such as the originally generated artifacts, a preserved checkpoint, a signed institutional digest, or an externally anchored Merkle root or block hash. Under this formulation, record-level verification no longer depends on permanent access to conversational content, but the tamper-evidence guarantee depends on the existence of a reference state that an adversary cannot silently rewrite.

### 3.4 Experimental environment and operational evaluation

The experimental evaluation was designed to analyze the framework's operational behavior under progressively increasing audited loads, different ledger structural configurations, and controlled cryptographic manipulation scenarios. The objective was not only to validate the alteration-detection capability but also to characterize the costs associated with evidence generation, lightweight hash-chained audit-ledger construction, and verification processes as transaction volume increases significantly.

The implementation was developed in Python, using specialized libraries for deterministic serialization, SHA-256 and HMAC-SHA-256 commitment generation, Merkle tree construction, and large-scale data processing. The full-corpus data-processing and storage experiments were executed on a workstation equipped with an Intel Core i9 processor, 64 GB RAM, Ubuntu 24.04 LTS, and Python 3.12. The repeated performance experiments reported in Tables 1–3, together with the numerical consistency audit, were executed on Windows 11 using Python 3.12.3 on a 13th Gen Intel(R) Core(TM) i9-13900HX workstation with 24 physical cores, 32 logical cores, and approximately 31.75 GB of RAM. All performance values reported in these tables correspond to five executions in the Windows 11 environment. The same serialization rules, cryptographic algorithms, and ledger-construction logic were maintained across scenarios.

**Table 1:** Repeated cryptographic audit-record generation, ledger construction, and full record-set verification performance on a fixed 139,258-record pilot audit set.

Observed metric	SHA-256	HMAC-SHA-256	Relative change in mean (%)
Audit-record generation throughput (records/s)	6,804.9515 (273.3189)	5,842.6104 (408.4802)	–14.1418
Operational ledger-stage throughput (records/s)	207,897.9769 (4,626.5327)	208,719.4096 (6,949.4942)	0.3951
Full record-set verification throughput (records/s)	135,589.6982 (5,101.0732)	135,606.5322 (3,147.0274)	0.0124
Successful verification runs	5/5	5/5	0.0000

Values are mean (SD) over five executions.

**Table 2:** Repeated operational latency and throughput across increasing audit loads.

Load	Total audit records	Total pipeline time (s)	Operational ledger-stage throughput (records/s)	Full record-set verification throughput (records/s)	Successful runs
10,000	49,258	7.393 (1.061)	107,508.042 (10,101.332)	136,433.450 (2,187.522)	5/5
50,000	89,258	14.035 (0.330)	159,984.079 (5,212.096)	131,989.959 (2,324.818)	5/5
100,000	139,258	23.031 (1.074)	211,834.429 (4,469.412)	136,656.735 (1,738.173)	5/5
250,000	289,258	50.275 (2.636)	299,382.457 (4,708.790)	142,165.946 (1,213.015)	5/5
500,000	539,258	95.090 (4.172)	202,371.588 (11,234.638)	102,345.913 (2,130.045)	5/5

Values are mean (SD) over five executions.

**Table 3:** Repeated block-size sensitivity metrics for in-memory block assembly and block-header chain validation on a fixed 139,258-record audit set.

Records per block	Block count	Ledger size (MiB)	In-memory block-assembly time (s)	Block-header chain-validation time (s)	In-memory block-assembly throughput (records/s)
100	1,393	0.710341	0.6441 (0.0085)	0.04203 (0.01941)	216,219.744 (2,822.041)
250	558	0.284359	0.6459 (0.0322)	0.01837 (0.01041)	216,014.992 (10,164.768)
500	279	0.142135	0.6320 (0.0095)	0.00682 (0.00242)	220,369.533 (3,310.998)
1,000	140	0.071665	0.6250 (0.0117)	0.00573 (0.00331)	222,868.923 (4,124.271)
2,000	70	0.035814	0.6271 (0.0095)	0.00258 (0.00126)	222,093.116 (3,301.479)
5,000	28	0.014322	0.6290 (0.0081)	0.00087 (0.00022)	221,410.342 (2,849.031)

Timing and throughput values are mean (SD) over five executions; block count is deterministic for each block size, while ledger size is reported as the mean serialized footprint across repeated executions.

The experimental dataset was obtained from LMSYS-Chat-1M, WildChat, and Chatbot Arena, resulting in a unified repository of 3,971,887 auditable units. Based on this, three groups of experiments were defined. The first analyzed the system's response to progressively increasing loads using scenarios of 10,000, 50,000, 100,000, 250,000, and 500,000 normalized interaction records. For comparability across load scenarios, each load also included the complete Chatbot Arena comparison set of 39,258 auditable comparison records. Consequently, the total audit-record counts were 49,258, 89,258, 139,258, 289,258, and 539,258 records, respectively. The term interaction load therefore denotes the number of normalized prompt-response interaction records selected for the scenario; it is not the number of original source conversations and should not be multiplied by the full-corpus average canonical yield ratio reported in Table 4. The full-corpus yield ratio characterizes the transformation from heterogeneous source records to auditable units, whereas the load experiment starts after normalization and varies the number of already canonical interaction records while holding comparison records constant. Each scenario was processed using the full audit pipeline, which recorded end-to-end execution time, per-stage latency, operational ledger-stage throughput, and full record-set verification throughput. End-to-end pipeline time includes canonical loading, audit-record generation, ledger construction, membership mapping, and verification. Operational ledger-stage throughput in this load experiment corresponds to the ledger simulator's internal timed section: reading the generated audit-record Parquet files, ordering records deterministically, partitioning them into blocks, computing Merkle roots from decision hashes, computing block hashes with previous-block links, creating membership rows, and writing the ledger headers as JSONL plus the membership map as Snappy-compressed Parquet. It excludes the upstream generation of audit-record commitments and excludes the downstream full record-set verification command. Full record-set verification denotes recomputation and validation over the complete record set generated in that load scenario.

**Table 4:** Canonical transformation yield and filtering metrics.

Dataset	Source records	Auditable units	Yield ratio	Filtered records	Filtering ratio (%)	Dataset contribution (%)
lmsys-chat-1m	1,000,000	1,989,603	1.9896	26,042	2.6042	50.0921
WildChat	837,989	1,943,026	2.3187	17,048	2.0344	48.9195
Chatbot_arena	33,000	39,258	1.1896	58	0.1758	0.9884
Interaction records total	1,837,989	3,932,629	2.1396	43,090	2.3444	99.0116
All canonical auditable units	1,870,989	3,971,887	2.1229	43,148	2.3062	100.0000

The second group of experiments evaluated the ledger's structural sensitivity by keeping the audited record set fixed at 139,258 records and varying only the maximum number of records per block. The configurations considered corresponded to block sizes of 100, 250, 500, 1,000, 2,000, and 5,000 records, enabling analysis of how the granularity of aggregation affects the number of blocks generated, the storage footprint, in-memory block-assembly throughput from precomputed commitments, and block-header chain-validation time. In this experiment, block-header chain-validation time includes only recomputation of block-header hashes and validation of previous-hash linkage and block order. It excludes Merkle-leaf generation, Merkle-root reconstruction from audit records, membership verification, audit-record commitment recomputation, data loading, and end-to-end verification of progressively larger workloads.

The third group of experiments was designed to detect deliberate manipulations across five attack categories: Merkle and decision-hash integrity, hash-chain linkage integrity, block ordering integrity, membership completeness, and dataset-membership consistency. A separate clean-ledger validation baseline was also executed, but it was not treated as a tampering category because no alteration was introduced. Each manipulated scenario was subsequently subjected to the complete cryptographic reconstruction procedure to evaluate its inconsistency-detection capability using only the evidence stored in the ledger.

To formally characterize the framework's behavior as audit load increases, scalability metrics were defined. The relative latency of scenario *s* is expressed in Equation 12:


$$\begin{array}{l} RL_{s}=\frac{T_{s}}{T_{ref}} \end{array}$$ (12)

where *T*_*s*_ denotes the total time required to execute the audit pipeline, and *T*_*ref*_ denotes the reference scenario.

The relative evolution of the ledger's build capacity is defined in Equation 13:


$$\begin{array}{l} RT_{s}=\frac{\Theta_{s}}{\Theta_{ref}} \end{array}$$ (13)

where Θ_*s*_ represents the operational ledger-stage throughput observed during lightweight audit-ledger construction in load scenario *s*.

The verification-to-ledger throughput ratio is expressed in Equation 14:


$$\begin{array}{l} VLR_{s}=\frac{\Gamma_{s}}{\Theta_{s}} \end{array}$$ (14)

where Γ_*s*_ corresponds to the throughput associated with full record-set verification in load scenario *s*. This ratio is a normalization diagnostic comparing two different throughput measures within the same load scenario, not an absolute measure of verification efficiency. It is calculated from the complete set of audit records generated for each workload and is therefore distinct from the block-header chain-validation time reported in the block-size sensitivity experiment.

Finally, the relative growth of the processed auditable volume is defined in Equation 15:


$$\begin{array}{l} SF_{s}=\frac{N_{s}}{N_{ref}} \end{array}$$ (15)

where *N*_*s*_ represents the total number of auditable records processed in the evaluated scenario and *N*_*ref*_ corresponds to the reference number. These metrics enable joint analysis of the pipeline's evolution over time, the operational ledger-stage build capacity, and the relative position of full record-set verification throughput as the volume of audited evidence increases. Block-size sensitivity was evaluated separately using a fixed 139,258-record set; consequently, its reported validation times refer to block-header chain validation under alternative block granularities and should not be compared directly with the full workload-verification throughput in Table 2.

### 3.5 Unified audit framework algorithm

The proposed audit framework comprises a sequence of interdependent transformations that link conversational data preparation, cryptographic commitment generation, lightweight hash-chained audit-ledger construction, and integrity validation. Within this process, the canonical representation determines the serialization of interactions, cryptographic commitments determine the construction of Merkle trees, and the resulting aggregated evidence subsequently participates in the validation procedures. Therefore, the system's final verifiability depends on the correct execution of all intermediate transformations that connect the original conversations to the resulting audit-ledger infrastructure.

To formalize the interactions among the various methodological components, Algorithm 1 summarizes the complete sequence of operations for constructing and validating the audit infrastructure. The procedure integrates the canonical preparation of conversational records, the generation of privacy-aware authenticated cryptographic commitments, the construction of blocks via Merkle aggregation, and the validation mechanisms for detecting structural alterations in the ledger.

Algorithm 1 **Canonical hash-chained auditability procedure.**

**Require:**  Public conversational datasets *D*, block size *b*, hash mode *alg*, and optional trusted reference checkpoint *C*, where *C* may be ∅
**Ensure:**  Audit ledger *B*, membership evidence *M*, verification status *s*, checkpoint status *c*
1:  Transform public source records into canonical auditable units *A*
   Extract valid prompt–response pairs or comparison records
   Discard structurally incomplete observations
   Assign dataset, conversation, turn, model, language, and timestamp fields
   Emit normalized units under the canonical schema
2:  Generate privacy-aware audit records *R*
   Serialize each canonical unit deterministically
   Compute prompt, response, metadata, and decision commitments
   Apply SHA-256 or HMAC-SHA-256 according to the selected audit mode
   Generate unique audit-record identifiers
   Store only commitments and operational traceability fields
3:  Construct lightweight hash-chained audit ledger *B*
   Order audit records deterministically
   Partition records into blocks of size *b*
   Compute Merkle leaves from record identifiers and decision commitments
   Generate Merkle roots for each block
   Compute block hashes using previous-block links
   Create membership evidence *M* for later verification
4:  Verify ledger integrity
   Reconstruct Merkle roots from *R* and *M*
   Recompute block hashes
   Validate previous-hash linkage
   Verify record membership associations
   Compare reconstructed values against stored ledger values
   If *C* ≠ ∅, compare the reconstructed checkpoint digest against *C*
5:  **if** no inconsistencies are observed and *C* ≠ ∅ and *C* matches the reconstructed checkpoint digest **then**
6:   *c* ← *matched*
7:   *s* ← *passed*
8:  **else if** *C* = ∅ and internal ledger values are mutually consistent **then**
9:   *c* ← *unavailable*
10:   *s* ← *internal*_*consistency*_*only*
11:  **else**
12:   *c* ← *failed*
13:   *s* ← *failed*
14:  **end if**
15:  **return** *B*, *M*, *s*, and *c*

The algorithm takes as input the public conversational sets, the aggregation configuration used during ledger construction, the cryptographic mechanism used to generate commitments, and an optional trusted reference checkpoint. The checkpoint may be a preserved original artifact, a signed institutional digest, or an externally anchored Merkle root or block hash; it may also be absent when the procedure is used only to evaluate internal consistency. The output corresponds to an audit ledger, membership evidence, and a verification status that distinguishes externally anchored tamper evidence from internal consistency only. Under this formulation, auditing no longer depends on the permanent storage of conversational content, but tamper evidence against ledger-wide rewriting requires comparison with *C*. If *C* = ∅, the algorithm can report only internal consistency of the supplied ledger artifacts and must not assert tamper evidence against an adversary capable of rewriting all stored ledger values. The procedure does not by itself prove original model ownership or the authenticity of upstream dataset provenance; those claims require independent source authentication, signed metadata, or institutional attestation outside the experimental ledger.

## 4 Results

### 4.1 Canonical preparation of auditable data

Table 4 presents the results of transforming the original conversational datasets into normalized, auditable units. From 1,870,989 source records, 3,971,887 auditable units were generated, representing an overall expansion of 2.12 times the original volume. This increase was due to each conversation being broken down into multiple prompt–response pairs, which could be audited individually, allowing the granularity of the analysis to exceed the original structure of the datasets.

The highest expansion factors were observed in WildChat and LMSYS-Chat-1M, with transformation ratios of 2.3187 and 1.9896, respectively. WildChat produced 1,943,026 auditable units from 837,989 original records, representing the highest transformation yield among the evaluated sources. This behavior indicates a higher conversational density per record, with a significant proportion of conversations containing multiple auditable exchanges. LMSYS-Chat-1M showed slightly lower expansion, yet it generated 1,989,603 auditable units and served as the primary source of evidence in subsequent experiments.

The relative contribution of both sources was highly balanced. LMSYS-Chat-1M contributed 50.09% of all auditable units generated, while WildChat accounted for 48.92%. Together, these two sets comprised 99.01% of the evidence used in the experimental evaluation. In contrast, Chatbot Arena contributed only 0.99% of the total, with 39,258 comparative records derived from 33,000 original observations. Although its volumetric contribution was considerably smaller, this dataset incorporated the dimension of human preferences between models, which was absent in the other datasets.

Filtering rates remained low across all three analyzed datasets. LMSYS-Chat-1M had a 2.60% removal rate, corresponding to 26,042 records discarded during validation. WildChat registered a 2.03% removal rate, equivalent to 17,048 excluded records. Chatbot Arena showed the lowest relative loss, with just 58 records removed and a 0.18% removal rate. Across the entire dataset, only 2.31% of the original records were discarded, indicating that most of the available information could be successfully converted into formats compatible with the audit layer.

The results shown in Figure 2A indicate that the expansion resulting from normalization was not uniform across the sources. While Chatbot Arena maintained a ratio close to 1 auditable unit per original observation (1.19×), the conversational sets exhibited growth factors of 2 or higher. This difference reflects the structural nature of the data: comparative records contain a single preference decision per observation, whereas complete conversations can generate multiple independent auditable events.

**Figure 2:**
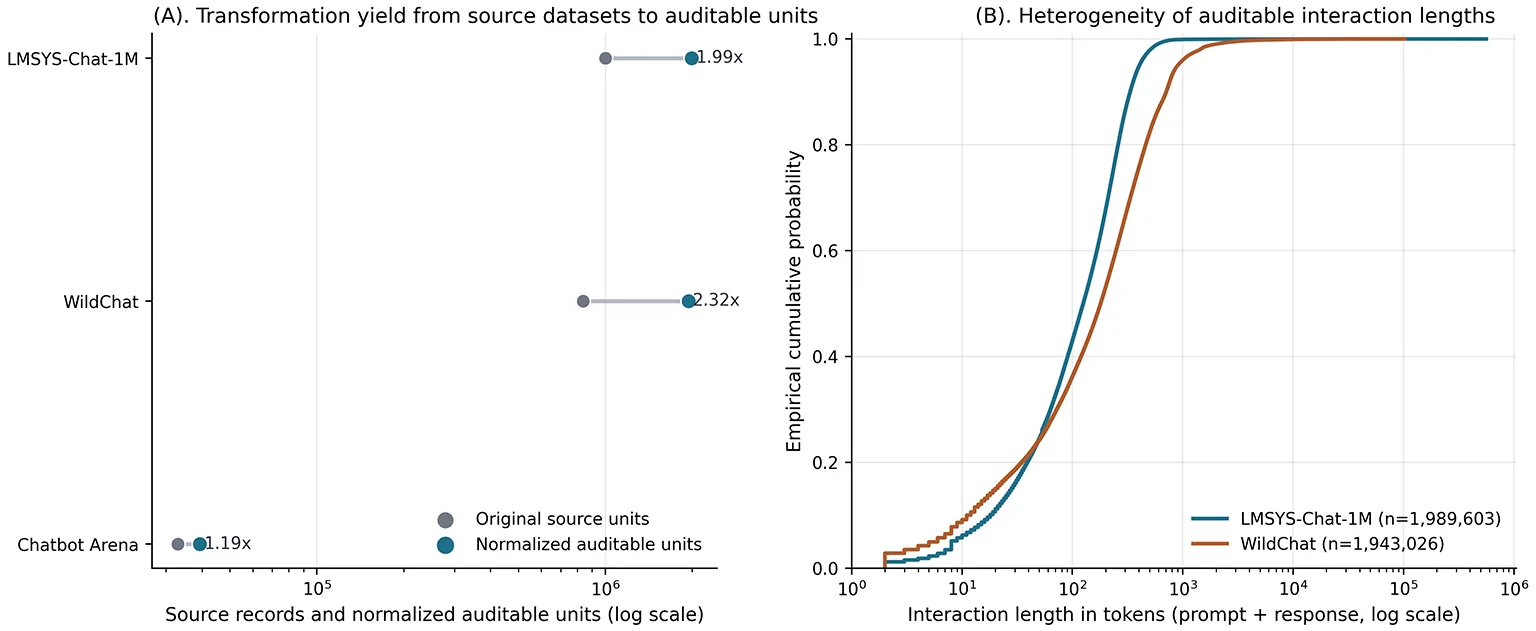
Canonical preparation of public LLM conversation datasets. **(A)** Compares the number of original source units with the normalized auditable units produced for LMSYS-Chat-1M, WildChat, and Chatbot Arena. **(B)** Reports empirical cumulative distribution functions for the token length of normalized prompt-response interactions in LMSYS-Chat-1M and WildChat using a logarithmic *x*-axis.

The heterogeneity of the normalized interactions is evident in Figure 2B. Both distributions exhibit wide dispersion in length, spanning several orders of magnitude in token count. LMSYS-Chat-1M shows a faster accumulation in the middle region of the distribution, reaching approximately 50% of observations with lengths shorter than those recorded by WildChat. In contrast, WildChat shows a more gradual cumulative transition between 100 and 1,000 tokens and a longer right tail, indicating greater dispersion and a relatively higher proportion of long conversations. This difference persists up to approximately 1,000 tokens, after which both distributions gradually converge toward cumulative probabilities close to unity.

### 4.2 Auditable privacy-preserving records

Table 5 summarizes the impact of hash-only representation on the storage requirements of the audit infrastructure. The normalized conversational repository used as a reference occupied 11,899.55 MiB. In the full-corpus HMAC-SHA-256 experiment, the 3,971,887 auditable units required 1,473.44 MiB as hash-only audit records, corresponding to an 87.62% reduction relative to the text-bearing normalized repository. The ledger itself was substantially smaller: block headers occupied 2.03 MiB, while the operational ledger plus membership structures required for record-level verification occupied 380.52 MiB.

**Table 5:** Storage footprint of the auditability framework in the full-corpus HMAC-SHA-256 experiment.

Observed metric	Size (MiB)	Auditable units covered	Storage reduction vs. off-chain (%)
Off-chain normalized repository	11,899.5515	3,971,887	0.0000
Hash-only audit records	1,473.4371	3,971,887	87.6177
Ledger block headers only	2.0255	3,971,887	99.9830
Ledger + membership structures	380.5151	3,971,887	96.8023

Storage footprints were measured from the physical byte size of the complete output artifacts generated during the full-corpus HMAC-SHA-256 experiment. The hash-only footprint corresponds to the sum of two Parquet files containing all 3,971,887 audit records: 1,525,558,227 bytes for standard interaction records and 19,452,515 bytes for comparison records. Both files encoded 64-character hexadecimal commitments and operational traceability fields using Snappy-compressed Parquet. The ledger used a block size of 1,000 auditable units. The ledger-header footprint was measured from the physical byte size of the JSONL block file, whereas the membership footprint was measured from the Snappy-compressed Parquet membership file; the operational-ledger value corresponds to the sum of both artifact groups. Sizes in MiB were calculated as total physical bytes divided by 1, 024^2^. Temporary logs, reports, summary tables, and auxiliary processing files were excluded from the storage totals.

The largest reduction was observed in the ledger block headers, whose total size was 2.03 MiB. This value should be interpreted strictly as the header footprint of the lightweight chain, not as the complete operational verification cost. When the membership structures required to locate records and reconstruct block-level evidence were included, the verifiable ledger footprint was 380.52 MiB, representing a 96.80% reduction relative to the original text-bearing repository. The hash-only audit-record files were larger because each row stores multiple 64-character hexadecimal commitments and operational traceability fields in Parquet format with Snappy compression. Standard interaction records include commitments for the prompt, response, metadata, moderation state, and aggregate decision hash, whereas comparative records additionally include separate commitments for both candidate responses.

Table 1 presents the operational behavior for generating auditable records, constructing the ledger, and performing full record-set verification under the SHA-256 and HMAC-SHA-256 configurations. Both hash modes were evaluated on the same pilot audit set of 139,258 records, composed of 100,000 standard interaction records and 39,258 comparison records. The reported values correspond to five repeated executions and are presented as mean and standard deviation.

Across the five repeated executions, HMAC-SHA-256 reduced audit-record generation throughput from 6,804.95 to 5,842.61 records per second on average, a 14.14% reduction consistent with the additional keyed authentication operation. Full record-set verification throughput was effectively unchanged: 135,589.70 records per second for SHA-256 and 135,606.53 records per second for HMAC-SHA-256. The mean difference of 0.0124% was smaller than the observed run-to-run dispersion and therefore does not provide evidence of a mechanical verification-speed advantage for either mode. Both configurations completed all five verification runs successfully. Tampering detection is reported separately in Table 6 because it evaluates induced manipulation scenarios rather than repeated hash-mode throughput.

**Table 6:** Aggregated tamper-detection performance and clean-ledger validation baseline on a fixed 139,258-record, 140-block audit ledger.

Validation category	Scenarios	Median validation time (ms)	IQR validation time (ms)	P95 validation time (ms)	Mean error signals
Merkle and decision-hash integrity	2	623.863	6.085	629.340	2.0
Hash-chain linkage integrity	2	611.152	11.222	621.252	2.0
Block ordering integrity	1	627.075	0.000	627.075	5.0
Membership completeness	2	609.348	21.060	628.302	3.5
Dataset-membership consistency	1	620.598	0.000	620.598	1.0
Clean ledger validation baseline	3	942.449	0.000	942.449	0.0

Figure 3A illustrates the progressive reduction in storage volume as conversational evidence is transformed into auditable artifacts. The most significant decrease occurs during the transition from the normalized text repository to hash-only records, where the size drops from 11,899.55 MiB to 1,473.44 MiB. Subsequently, the representation is further reduced at the audit-ledger layer: the operational ledger plus membership mapping occupies 380.52 MiB, and the block headers alone occupy 2.03 MiB. This pattern confirms the framework's ability to decouple verifiability from the permanent storage of conversational content while preserving the auxiliary structures needed for record-level verification.

**Figure 3:**
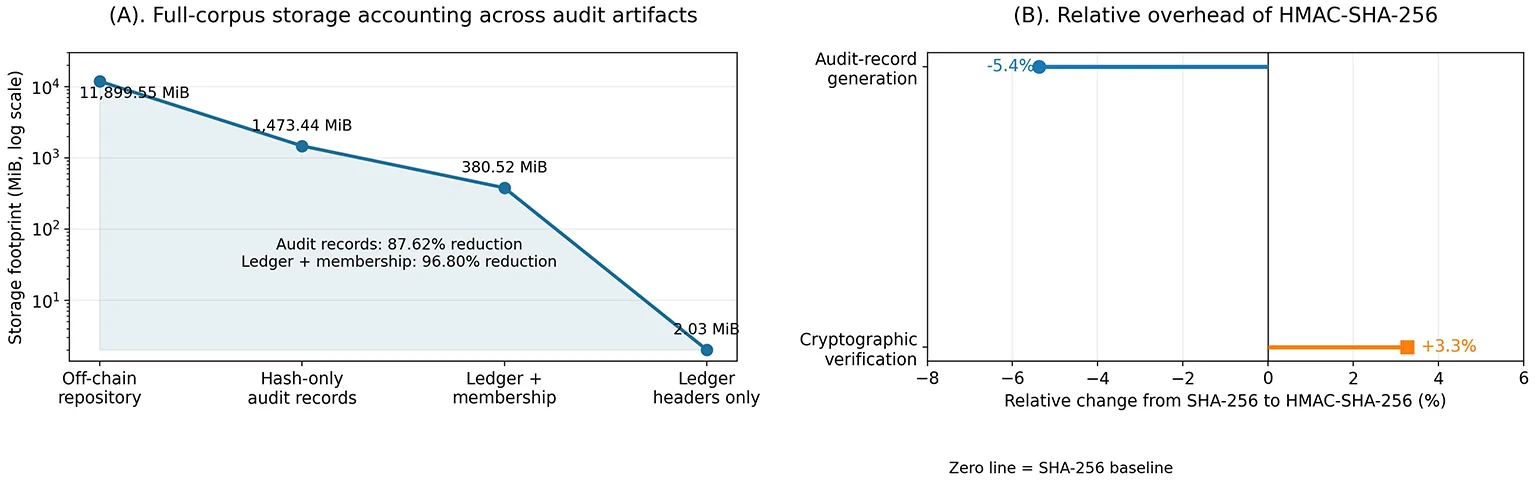
Privacy-preserving audit artifacts and hashing performance. **(A)** Reports the full-corpus storage footprint of the normalized off-chain repository, hash-only audit records, ledger plus membership structures, and ledger block headers using a logarithmic reduction curve. **(B)** Reports the mean relative change from SHA-256 to HMAC-SHA-256 for audit-record generation and full record-set verification throughput measured on the same 139,258-record pilot audit set over five executions.

Figure 3B shows that the main operational cost of HMAC-SHA-256 appears during audit-record generation, where keyed commitments must be produced from the canonical fields. Full record-set verification remains practically unchanged within the observed dispersion. Thus, incorporating authenticated commitments introduces a measurable generation penalty but does not materially alter the verification capacity of the evaluated pilot ledger.

To make the empirical positioning of the proposed framework explicit, Table 7 compares it with closely related blockchain-audit approaches discussed in the literature. The comparison is qualitative because the prior systems target different domains, datasets, and threat models; direct numerical benchmarking would therefore be misleading. Instead, the table evaluates whether each approach operationalizes the components required by the present study: construction of auditable units, content-minimizing commitments, ledger-level verification, record-level membership, induced tampering tests, and large-scale conversational evaluation.

**Table 7:** Qualitative comparison with closely related blockchain-audit frameworks.

Framework	Auditable unit	Privacy model	Verification evidence	Tampering evaluation	Scale/domain
Zhou (2025)	Securities trade	Zero-knowledge and permissioned blockchain	Transaction and compliance evidence	Trading-system security metrics	Financial trading
Davies (2024)	Merkelized transaction fields	Selective field verification and redaction	Merkle proofs over fields	Protocol-level privacy and scalability analysis	Blockchain data
Li et al. (2026)	LLM model update	Hash-chain audit trail	Update-level compliance verification	Model-update audit validation	LLM adaptation workflow
Li et al. (2024)	Supply-chain event	Privacy-preserving traceability model	Supply-chain provenance evidence	Traceability and system evaluation	Supply chain
This study	Normalized LLM turn or comparison	SHA-256/HMAC-SHA-256 commitments with off-chain content	Merkle roots, block hashes, and membership mapping	Five induced attack categories plus clean-ledger baseline	3.97 million conversational auditable units

The comparison shows that the proposed framework does not merely replace content with hashes; it adds the missing canonicalization layer needed for heterogeneous LLM conversations before ledger-based verification can occur. Compared with transaction-centered or model-update-centered systems, the distinctive element is transforming irregular prompt-response and comparison data into stable, auditable units, followed by full-corpus storage accounting and explicit record-level membership verification.

### 4.3 Cryptographic integrity and tamper detection

Table 6 summarizes the behavior observed in the controlled functional validation of manipulated ledger artifacts and in the clean-ledger validation baseline. The tampering experiment used the same 139,258-record audit set and 140-block ledger as the pilot configuration. Eight manipulated scenarios spanning five predefined categories were detected, with no false acceptances in this controlled experiment. This behavior was consistent across local alterations, modifications of individual hashes, and audited decisions, as well as in structural manipulations that affected block chaining or the integrity of membership proofs. The clean-ledger row is reported separately as a baseline validation case, where the expected result is successful acceptance of an untampered ledger rather than detection of an attack.

The most visible differences between manipulated categories were observed in the latency required to detect the alteration. The lowest descriptive median detection times were recorded for attacks on membership integrity, at 609.35 ms, followed by hash chaining attacks at 611.15 ms. Alterations to Merkle integrity and decision hashes required 623.86 ms, while modifications related to block ordering had the highest observed median among tampering cases: 627.08 ms. The total range between the lowest and highest manipulated-category medians was less than 18 ms within the controlled scenarios.

Temporal dispersion is reported descriptively because some categories contain only one or two manipulated scenarios. Attacks related to membership completeness registered the largest interquartile range, at 21.06 ms, followed by hash chaining attacks, at 11.22 ms. In contrast, alterations in block ordering and membership consistency showed zero dispersion values because there was only one experimental scenario per category. The 95th percentile values should therefore be read as descriptive summaries of the controlled validation cases, not as population-level estimates of a latency distribution.

The clean-ledger validation baseline required a higher median time, 942.45 ms, because it measures successful reconstruction and acceptance of an untampered ledger rather than early rejection after an induced inconsistency. In the tampering scenarios, verification can stop once the first inconsistency is detected in the reconstructed evidence chain. In the clean baseline, by contrast, the procedure must complete the validation path without encountering a failure signal. The higher value therefore does not indicate that tampering is easier to verify than an intact ledger; it reflects the difference between full successful validation and fault-triggered rejection. This baseline was computed from the clean ledger before mutation, using the same verification procedure applied to the tampered cases. It confirmed sequential hash-chain validation and Merkle-membership reconstruction with zero error signals, providing the acceptance reference against which the induced tampering scenarios were interpreted.

The cumulative latency distribution shown in Figure 4A confirms this behavior. Approximately half of the detections occur before 621.5 ms, while the 95th percentile is reached at approximately 630.2 ms. The small difference between these two values indicates that most observations are concentrated within a narrow time window, with no long tails or isolated episodes of significant degradation during the validation process.

**Figure 4:**
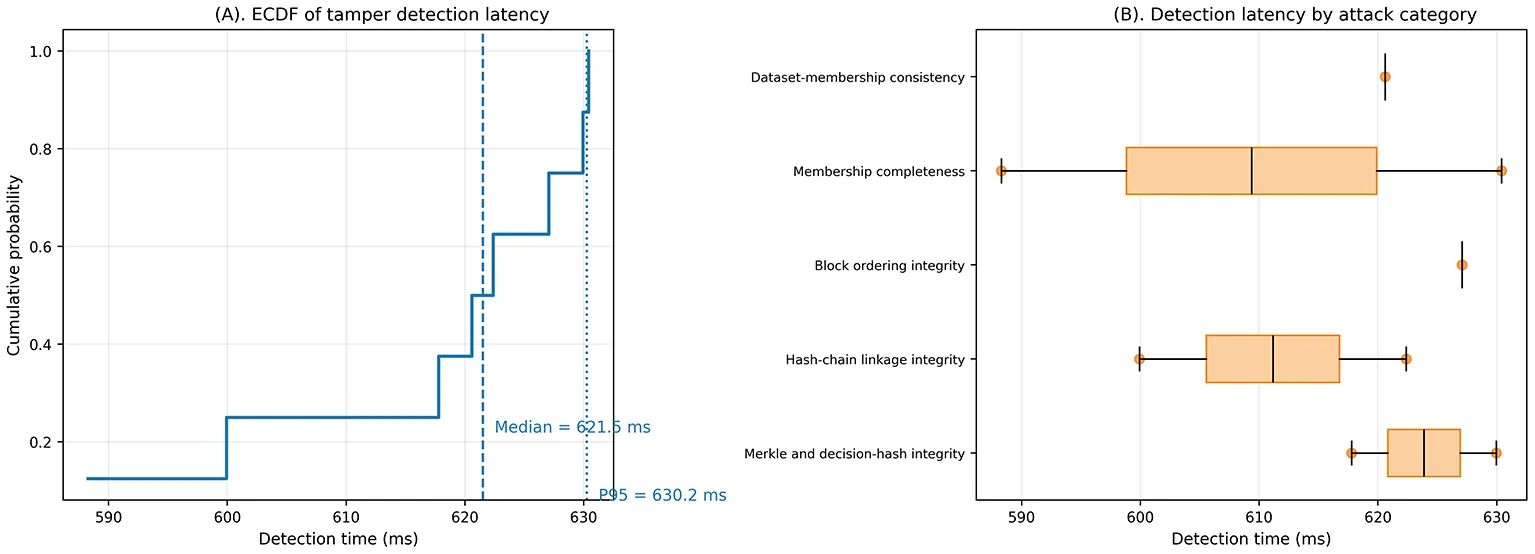
Controlled tampering detection results. **(A)** Reports the empirical cumulative distribution of detection latency across manipulated ledger scenarios. **(B)** Summarizes detection-time dispersion by attack category using boxplots with observed scenario points overlaid.

Figure 4B allows comparison of the time dispersion across the attack categories. Scenarios associated with Merkle integrity and decision hashes exhibit the most compact distribution around 624 ms. At the same time, manipulations related to membership completeness show considerably greater variability, ranging from approximately 589 ms to about 630 ms. Hash chaining attacks occupy an intermediate position, mainly occurring between 600 and 622 ms. Despite these differences in dispersion, all categories remain within the same operational detection range.

Attacks on block ordering generated the highest average number of error signals, at 5.0, surpassing membership completeness attacks (3.5) and alterations to cryptographic integrity or hash chaining (2.0). In contrast, cross-dataset consistency scenarios and membership tests triggered only one error signal per event. This difference shows that structural alterations of the ledger produce a wider spread of inconsistencies than modifications localized to individual records.

### 4.4 Operational performance under increasing load

The progressive increase in the audited workload led to a sustained increase in the total pipeline execution time. As shown in Table 2, each load configuration was executed five times and is reported as mean and standard deviation. The smallest scenario, consisting of 49,258 auditable records, required 7.39 s on average to complete the measured processing stages, while the largest, with 539,258 records, required 95.09 s. All scenarios completed the ledger construction and verification procedures successfully in all five executions. Because each load scenario includes the same 39,258 Chatbot Arena comparison records, the proportion of comparison records is highest in the smaller workloads and decreases as interaction records increase. Therefore, these measurements characterize scalability with respect to total auditable records under the stated load construction, not a constant record-type mixture experiment.

The trend observed in Figure 5A indicates an approximately linear increase in mean total pipeline time with respect to the number of generated audit records. A least-squares linear fit between total audit records and mean total pipeline time yielded *R*^2^ = 0.999960, with a slope of approximately 1.80 × 10^−4^ s per record, or 17.96 s per additional 100,000 records. Thus, the observed increase reflects predictable scaling with audited volume rather than an acceleration threshold after 250,000 records.

**Figure 5:**
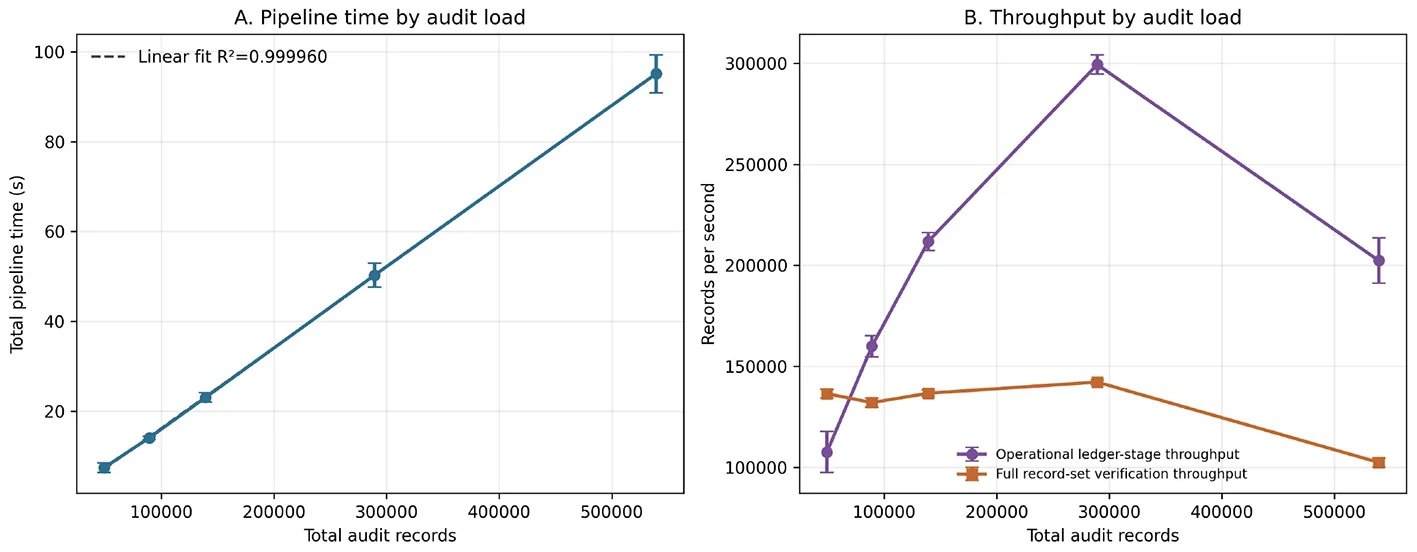
Operational performance under increasing audit loads. **(A)** Plots mean total pipeline time with standard-deviation bars across evaluated load scenarios. **(B)** Compares mean operational ledger-stage throughput and mean full record-set verification throughput with standard-deviation bars across the same scenarios.

The repeated measurements show that operational ledger-stage throughput increases from the 10,000- to the 250,000-interaction scenario and then decreases in the 500,000-interaction scenario. This non-monotonicity is visible in Figure 5B and should be interpreted as an empirical runtime effect rather than as a structural break in the ledger design. The mean total pipeline time remains almost perfectly linear with total audit-record volume despite the changing record-type composition and the throughput variation at the largest workload. Full record-set verification throughput remains comparatively stable from 10,000 to 250,000 interactions, between approximately 132k and 142k records/s, before decreasing to approximately 102k records/s in the largest scenario. This metric corresponds to complete verification over the generated record set in each load scenario, not to the block-header chain-validation timings reported separately in Table 3.

The verification success rate remained at 1.0 in all experimental scenarios. No cryptographic failures were observed either during the validation of the auditable records or during the reconstruction of the integrity tests used in the load experiments. The observed throughput and latency values indicate that increasing audit volume primarily affects cumulative processing time. Verification capacity remained comparatively stable through the 250,000-interaction scenario but decreased at the largest evaluated workload, indicating the emergence of system-level resource effects at that scale.

### 4.5 Sensitivity by block size

The quantitative results obtained for the different grouping configurations are presented in Table 3. Increasing the block size from 100 to 5,000 records resulted in a progressive reduction in the total number of generated blocks, from 1,393 to 28. In parallel, the ledger's serialized size decreased from 0.7104 MiB to 0.0143 MiB. Since the total volume of audited records remained constant across all scenarios, the observed differences reflect only the effect of grouping granularity on the resulting ledger structure. The storage column uses the same serialized JSONL block-header representation and binary MiB unit used for the full-corpus storage accounting in Table 5.

The combined evolution of block count and ledger size is shown in Figure 6A. The most pronounced reduction is concentrated in the first configurations evaluated. When the block size increases from 100 to 250 records, the number of blocks decreases from 1,393 to 558, while the ledger size decreases from 0.7104 MiB to 0.2844 MiB. A similar behavior is observed when increasing the number of records per block from 250 to 500, where both metrics again decrease to approximately half their previous values.

**Figure 6:**
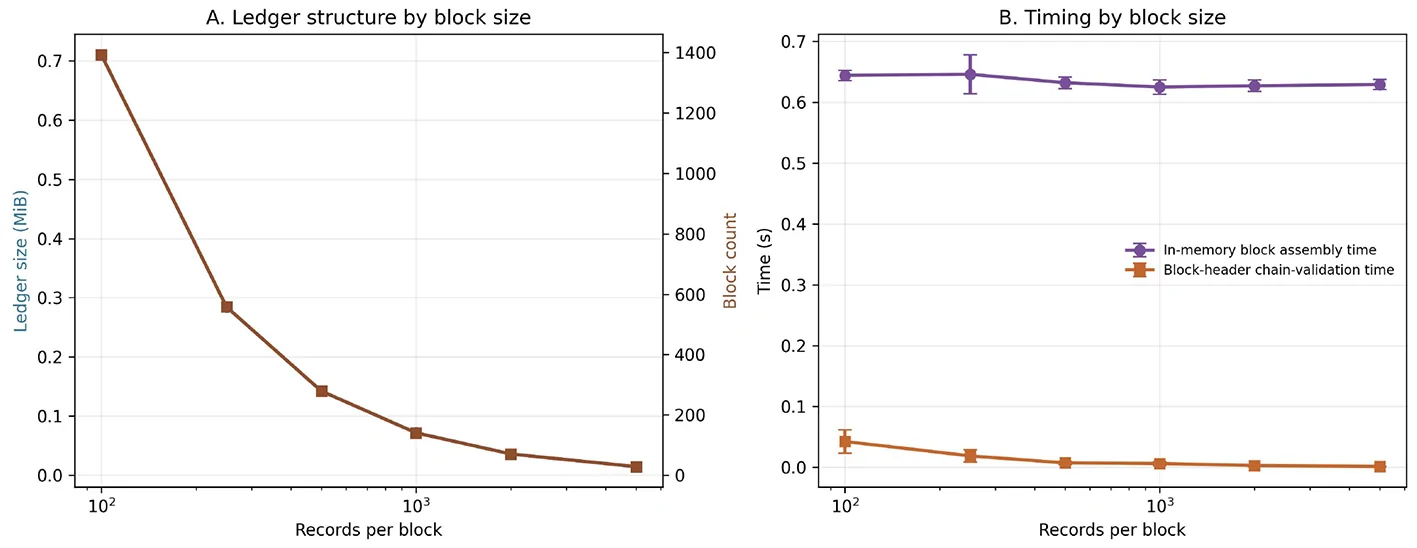
Block-size sensitivity of the audit ledger. **(A)** Reports mean serialized ledger size and deterministic block count across records-per-block settings for a fixed 139,258-record audit set. **(B)** Compares mean in-memory block-assembly time and mean block-header chain-validation time with standard-deviation bars across the same parameter values.

Starting at 1,000 records per block, the slope of both curves begins to decrease. The ledger size drops from 0.0717 MiB with 1,000-record blocks to 0.0358 MiB with 2,000-record blocks, and subsequently to 0.0143 MiB with 5,000-record blocks. Although the reduction remains visible, the absolute changes between consecutive configurations are considerably smaller than those observed in the early stages of the experiment. The same behavior is evident in the total number of blocks, indicating a region of diminishing returns, where further increases in block size yield progressively smaller structural benefits.

The simultaneous decrease in the number of blocks and the storage footprint indicates a virtually proportional relationship between the two variables. While the number of blocks decreases approximately 50-fold between the extreme configurations, the ledger size decreases by a similar proportion. This behavior indicates that a significant portion of total storage is associated with headers, chaining structures, and block metadata, and that this amount decreases directly with increasing ledger fragmentation.

In-memory block-assembly times showed considerably less variation than that observed in the structural metrics. Mean values ranged from 0.6250 s to 0.6459 s across all evaluated configurations, while mean throughput remained close to 216k–223k records per second. These values differ from the operational ledger-stage throughput in Table 2 because the block-size experiment isolates repeated ledger assembly from a fixed audit set, whereas the load experiment measures the ledger stage inside the broader pipeline context after regenerating workload-specific audit records. The difference therefore reflects different timed scopes, not contradictory measurements of the same operation.

Figure 6B shows that mean in-memory block-assembly times remain practically constant across the evaluated range. The proximity of the experimental points indicates that this isolated stage is relatively insensitive to changes in clustering granularity. The block-header chain-validation curve exhibits clearly distinct behavior because this experiment evaluates only recomputation of block-header hashes and validation of previous-hash linkage and block order over the fixed 139,258-record audit set. The configuration of 100 records per block required 0.04203 s on average to complete block-header chain validation, while the 5,000-record configuration reduced this time to 0.00087 s. These values should not be interpreted as full record-set verification times; they exclude Merkle-leaf generation, Merkle-root reconstruction from audit records, membership verification, audit-record commitment recomputation, and data loading.

The greatest absolute reduction in block-header chain-validation time occurs between 100 and 250 records per block because the number of block headers decreases sharply in that interval. From this point, the downward trend continues, although with progressively smaller differences between consecutive configurations. The distribution of experimental points shows a constant decrease over the entire evaluated range, with no intermediate increases or abrupt changes in behavior.

### 4.6 Scalability by audit load

The results obtained for the different levels of audited load are presented in Table 8. The increase in interaction volume led to simultaneous increases in both the total number of auditable records and the number of ledger blocks generated. The smallest load generated 49,258 auditable records across 50 blocks, while the largest load generated 539,258 records across 540 blocks. During this increase, mean relative latency rose from 1.0 to 12.8616. Mean relative ledger throughput increased up to the 250,000-interaction scenario and then decreased in the 500,000-interaction scenario, indicating that the largest workload did not continue the fixed-cost amortization observed in the middle scenarios.

**Table 8:** Load-based scalability metrics.

Interaction load	Total audit records	Ledger blocks	Relative latency	Relative ledger throughput	Verification-to-ledger throughput ratio	Scalability factor
10,000	49,258	50	1.0000	1.0000	1.2691	1.0000
50,000	89,258	90	1.8983	1.4881	0.8250	1.8121
100,000	139,258	140	3.1151	1.9704	0.6451	2.8271
250,000	289,258	290	6.8001	2.7847	0.4749	5.8723
500,000	539,258	540	12.8616	1.8824	0.5057	10.9476

The evolution of relative latency is shown in Figure 7A. Because relative latency is normalized by the baseline scenario, its mean values increase from 1.0 to 12.8616 as the audited record count increases from 49,258 to 539,258. This pattern is consistent with the near-linear end-to-end timing observed in Table 2; it does not indicate a separate acceleration regime. The total audit-record volume increased by a factor of 10.9476, while mean relative latency increased by a factor of 12.8616.

**Figure 7:**
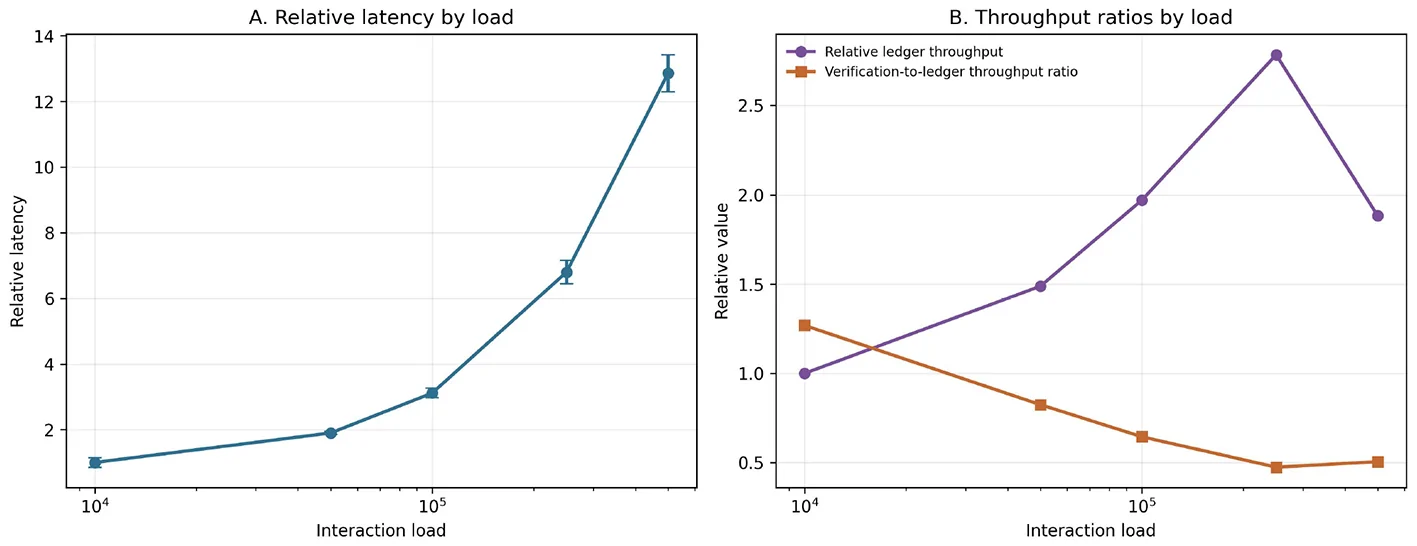
Load-based scalability of the audit pipeline. **(A)** Reports relative latency as interaction load increases. **(B)** Compares relative ledger throughput and the verification-to-ledger throughput ratio across the same load scenarios.

The larger absolute differences between later points are therefore a consequence of comparing wider workload intervals rather than evidence of nonlinear degradation. The increase from 250,000 to 500,000 input interactions covers the largest change in generated audit records, from 289,258 to 539,258, and correspondingly produces the largest absolute increase in total runtime. Interpreted against total audit records, however, the pipeline follows an almost linear timing profile.

This distinction is important because the evaluated load scenarios are not equally spaced in generated records. Consequently, visual separation between points in Figure 7A should be read together with the scalability factor in Table 8. The results support predictable growth of cumulative runtime and do not show an abrupt performance break within the evaluated range.

The relationship between relative throughput and the verification-to-ledger throughput ratio is shown in Figure 7B. Mean operational ledger-stage throughput increases from 1.0 in the baseline scenario to 1.4881, 1.9704, and 2.7847 in the 50,000-, 100,000-, and 250,000-interaction scenarios, respectively, before decreasing to 1.8824 in the largest scenario.

This pattern indicates that operational ledger-stage throughput benefits from fixed-cost amortization in the middle workloads but does not increase monotonically across the entire evaluated range. The largest scenario remains substantially above the baseline throughput, but the drop relative to the 250,000-interaction scenario shows that system-level effects become visible at the highest tested volume.

The verification-to-ledger throughput ratio decreases from 1.2691 in the baseline scenario to 0.4749 in the 250,000-interaction scenario, then increases slightly to 0.5057 in the largest scenario. This behavior reflects the fact that the ratio compares two throughputs with different scaling behavior; it is therefore a normalization diagnostic rather than an absolute efficiency measure. Full record-set verification throughput itself remains between approximately 132k and 142k records/s up to the 250,000-interaction scenario and decreases to approximately 102k records/s in the largest scenario.

## 5 Discussion

The literature review has identified the need to audit AI systems but has not yet fully resolved the issue of verifiable traceability of LLM interactions at scale. Mökander et al. (2024) proposes a layered audit useful for assigning responsibilities, although they do not operationalize cryptographic logging for each interaction. Li et al. (2026) makes progress with hash chains for auditable LLMs, but the problem of off-chain canonical preparation remains less addressed. Complementary studies further position these results. Bhumichai et al. (2024) frames AI-blockchain convergence as a still-fragmented promise; Widder et al. (2024) cautions that the openness of AI systems is often incomplete; and Han et al. (2025) shows that privacy and auditability can coexist through cryptographic data separation. In this context, our central finding is that auditability depends not only on adding blockchain data but also on producing verifiable canonical units before anchoring them.

The evidence is especially strong because it shows that heterogeneous public conversations can be transformed into an off-chain basis complete enough for auditing. LMSYS-Chat-1M produced 1,989,603 normalized interactions from 1,000,000 conversations; WildChat produced 1,943,026 normalized interactions from 837,989 records; and Chatbot Arena generated 39,258 comparison records from 33,000 comparisons. The total of 3,971,887 auditable units confirms that turn-by-turn extraction is not a methodological detail, but rather the mechanism that converts irregular conversational data into computable evidence. The ECDF of lengths reinforces this point: the proposed auditability is not limited to short or uniform conversations but encompasses a range of interaction scales.

The storage result is one of the most revealing of the study, but it must be interpreted as storage accounting rather than conventional compression. Compared to an off-chain normalized repository of 11,899.55 MiB, the full-corpus HMAC-SHA-256 audit records occupied 1,473.44 MiB, while ledger block headers occupied 2.03 MiB. When we included the membership mapping required for operational record-level verification, the ledger footprint was 380.52 MiB. This separation relocates evidentiary weight: sensitive content remains off-chain, hash-only audit records retain reproducible commitments and traceability fields, and the lightweight ledger stores aggregate evidence for verification. That distinction is crucial for LLMs, because publishing prompts or full responses would be incompatible with privacy, responsible data reuse, and institutional governance.

The comparison between SHA-256 and HMAC-SHA-256 shows that the additional privacy layer does not compromise operational feasibility, but the privacy claim must be interpreted together with the key-custody model defined in the methodology. Across five repeated executions, HMAC-SHA-256 reduced audit-record generation throughput from 6,804.95 to 5,842.61 records per second on average, a relative penalty of 14.14%, while full record-set verification throughput remained effectively unchanged, from 135,589.70 to 135,606.53 records per second. This evidence supports a defensible design decision for scenarios where revealing direct content hashes can enable inference or dictionary attacks, provided the deploying institution governs key generation, storage, rotation, and auditor access.

The controlled manipulation experiments support the functional verifiability claim without presenting the small scenario groups as a population-level robustness study. Across eight induced manipulations spanning five attack categories- Merkle and decision-hash integrity, hash-chain linkage integrity, block ordering integrity, membership completeness, and dataset-membership consistency- the system achieved a detection rate of 1.0 and a false acceptance rate of 0. The clean-ledger baseline was evaluated separately and produced zero error signals. The descriptive latencies for manipulated categories, between 588 and 630 ms, indicate that the implemented verification procedure detects the predefined inconsistencies within an operationally reasonable window in this controlled setting.

Sensitivity to block size and scalability under load show that the framework maintains predictable performance as the audited record volume and block-size configuration vary. With loads ranging from 10k to 500k normalized interaction records plus the fixed Chatbot Arena comparison set, mean pipeline time increased from 7.393 to 95.090 s. The near-linear relationship between total audit records and mean cumulative pipeline time indicates that the dominant cost grows predictably with the number of generated records rather than with an abrupt ledger-construction bottleneck. Therefore, the most innovative contribution is not to affirm that blockchain can audit LLMs in the abstract, but to demonstrate experimentally that a hash-only, off-chain, and canonical architecture can jointly support privacy-aware commitment representation, storage reduction, manipulation detection relative to trusted checkpoints, and scalable verification within the same chain of evidence.

## 6 Conclusion

This work proposes and experimentally validates an auditability framework for LLM-generated decisions that shifts the emphasis from simply observing outputs to building verifiable evidence. The main contribution lies not in incorporating blockchain as an external recording component but in demonstrating that public conversations from LLM systems can be transformed into off-chain canonical units, protected by cryptographic commitments and subsequently verifiable using a lightweight ledger layer. This architecture addresses a critical need in contemporary AI auditing: maintaining traceability without exposing sensitive conversational content.

The results confirm that the canonical stage constitutes the technical core of the framework. The transformation of LMSYS-Chat-1M, WildChat, and Chatbot Arena yielded 3,971,887 auditable units, demonstrating that heterogeneous sources can become a common basis for subsequent verification. This evidence is relevant because the auditability of LLMs cannot rely solely on the original logs, whose structure, length, and granularity vary across platforms. The proposed framework demonstrates that verifiable traceability requires, first and foremost, a standardized, stable, and reproducible representation of the audited event.

The evaluation also shows that privacy and verifiability are not incompatible goals. By separating off-chain content and on-chain evidence, the system represented the 11,899.55 MiB text-bearing repository as 1,473.44 MiB of full-corpus HMAC-SHA-256 audit records, 2.03 MiB of ledger block headers, and 380.52 MiB for the ledger plus membership structures needed for record-level verification. The use of HMAC-SHA-256 added key protection with a moderate generation penalty, without compromising full record-set verification in the evaluated scenarios. Consequently, the study provides a technically defensible alternative for scenarios where publishing direct prompts, responses, or hashes could compromise privacy, security, or institutional governance.

The framework's functional validity is further supported by its detection of induced manipulations and its scalability. All eight induced manipulations spanning the five predefined categories, Merkle and decision-hash integrity, hash-chain linkage integrity, block ordering integrity, membership completeness, and dataset-membership consistency, were detected with no false acceptances in this controlled experiment, while the clean-ledger baseline validated the unmodified chain with zero error signals. Load experiments further showed that lightweight audit-ledger construction maintains high performance even as record volume increases, with near-linear total pipeline timing relative to audit-record volume. These findings allow us to assert that the proposal is not limited to a conceptual model: it constitutes an experimental chain of evidence connecting data preparation, commitment generation, ledger construction, verification, and performance evaluation.

In terms of scope, the framework was evaluated on public datasets and a lightweight hash-chained audit-ledger architecture, so it is not intended to replace distributed consensus infrastructures or to solve governance, authorization, original provenance authentication, or full institutional key-management problems on its own. The HMAC-SHA-256 mode specifies the cryptographic dependency on a controlled key and its consequences for verification. Production deployment would still require operational controls for custody, rotation, revocation, authorized auditor access, and external anchoring of selected ledger checkpoints when adversaries may control stored ledger artifacts. These limitations do not weaken the core contribution; they define it precisely. The study's value lies in establishing an auditable, reproducible, and extensible foundation on which more complex institutional mechanisms can be integrated.

The work demonstrates that the auditability of LLMs can be formulated as a measurable experimental problem rather than merely a regulatory requirement. The proposal offers a concrete path to transform conversational interactions into verifiable, privacy-aware, and scalable evidence. This outcome opens a line of work for independent audits, the traceability of generative decisions, regulatory compliance, and the forensic evaluation of LLM systems deployed in real-world contexts.

## Data Availability

The datasets presented in this article were conducted using publicly available conversational datasets. The datasets can be accessed through their original repositories: WildChat-1M: https://huggingface.co/datasets/allenai/WildChat and LMSYS Chat-1M: https://huggingface.co/datasets/lmsys/lmsys-chat-1m. Requests to access the datasets should be directed to william.villegas@udla.edu.ec.
